# Functional Consequences of the Disturbances in the GABA-Mediated Inhibition Induced by Injuriesin the Cerebral Cortex

**DOI:** 10.1155/2011/614329

**Published:** 2011-05-31

**Authors:** Barbara Imbrosci, Thomas Mittmann

**Affiliations:** Institute of Physiology and Pathophysiology, Medical Center of the Johannes Gutenberg University, Duesbergweg 6, 55128 Mainz, Germany

## Abstract

Cortical injuries are often reported to induce a suppression of the intracortical GABAergic inhibition in the surviving, neighbouring neuronal networks. Since GABAergic transmission provides the main source of inhibition in the mammalian brain, this condition may lead to hyperexcitability and epileptiform activity of cortical networks. However, inhibition plays also a crucial role in limiting the plastic properties of neuronal circuits, and as a consequence, interventions aiming to reestablish a normal level of inhibition might constrain the plastic capacity of the cortical tissue. A promising strategy to minimize the deleterious consequences of a modified inhibitory transmission without preventing the potential beneficial effects on cortical plasticity may be to unravel distinct GABAergic signaling pathways separately mediating these positive and negative events. Here, gathering data from several recent studies, we provide new insights to better face with this “double coin” condition in the attempt to optimize the functional recovery of patients.

## 1. Introduction

Cortical injuries are one major cause of death and permanent disabilities worldwide. In the attempt to ameliorate the survival rate and the postlesion rehabilitation of patients, researchers have developed several animal models of cortical injury to reproduce different aspects of this pathological condition.

In particular, a great effort has been dedicated in the investigation of the physiological disturbances spreading in the surrounding uninjured tissue and sometimes even in remote brain areas [1].

Even though these lesion-induced functional alterations might notably differ depending on many factors, such as the nature of the insult (cerebrovascular rather than traumatic), the extent of the damage and the cortical structures affected, some pathophysiological events have been systematically reported following many different experimental models of cortical lesion.

Interestingly, one of the most frequently observed functional change postlesion is a reduction in the GABA-mediated inhibition which, therefore, seems to be (with some degrees of variability) a general phenomenon taking place as a consequence of a massive neuronal death.

Because a deficit in the GABAergic transmission might easily compromise the delicate balance between excitatory and inhibitory neurotransmission [2] this lesion-induced phenomenon has been strongly implicated in the generation of hyperexcitable cortical networks [3] and in the genesis of epileptic events often observed after brain injuries [4, 5].

However, the inhibitory action of GABA is going far beyond the control of the excitability of neuronal networks. The temporal and spatial precise release of GABA can also guarantee high specific responses of cortical neurons [6, 7]. Moreover, the GABAergic transmission has a fundamental role in controlling the plastic capacity of cortical networks. On this concern, different studies indicate that if the strength of the GABA-mediated inhibition is falling below a certain threshold, the plastic properties of the cortical networks will be augmented, sometimes even to levels similar to those observed during the critical period for plasticity [8–10].

In light of these findings, the impaired inhibitory transmission observed postlesion might not be only a deleterious process but, by enhancing the plastic capacity of the cortex, could also promote the functional reorganization of the surrounding uninjured cortical tissue contributing to the functional recovery from the lesion-induced neurological deficits. 

The injury-induced reduction of inhibition may, therefore, share both detrimental and beneficial effects. 

Unraveling distinct GABAergic signaling pathways separately mediating these positive and negative events could be extremely helpful in the design of a more effective postlesion rehabilitation therapy. 

In the attempt to provide new insights to better face with this “double coin” condition, in this paper we will discuss several studies which documented a reduced and/or an altered GABAergic transmission as a consequence of a lesion in the cerebral cortex, and most importantly, we will try to explain how and through which cellular mechanisms the altered GABAergic transmission could influence functions, excitability, and plasticity of cortical networks.

## 2. Physiology of GABAergic Signaling

The GABA receptors are divided into 2 classes: GABA_A_ receptors (GABA_A_Rs) and GABA_B_ receptors (GABA_B_Rs) (previously GABA_C_Rs were considered to form a third separated class; however, because of their strong structural and functional similarity to GABA_A_Rs, they are today classified as a subfamily of GABA_A_Rs).

**GABA_A_Rs**

GABA_A_Rs belong to the cys-loop superfamily of ligand-gated ion channels and mediate fast synaptic inhibition in the central nervous system (CNS).

GABA_A_Rs are heteropentameric structure composed by distinct types of subunit. In the mammalian brain, the majority of synaptic GABA_A_Rs are formed by two *α*, two *β* and one *γ* subunit.

Although many different *α*, *β*, and *γ* subunits have been identified (*α* 1–6, *β* 1–3, *γ* 1–3), in the CNS defined combinations of subunits are more frequently found (the most abundant combinations are *α*1, *β*2, *γ*2; *α*2, *β*3, *γ*2; *α*3, *β*3, *γ*2) [11]. 

Importantly, the combination of these subunits can determine the localization and the functional properties of the receptors. To mention a peculiar example, GABA_A_Rs in which the *γ* subunit has been replaced with the *δ* subunit are exclusively found extrasynaptically [12], are activated by low concentrations of GABA and they display a reduced desensitization [13, 14]. Thanks to these properties *δ* subunit-containing GABA_A_Rs are ideally suited to mediate tonic inhibition [15]. 

GABA_A_Rs are selectively permeable to Cl^−^ and to a less extent to HCO_3_^−^ [16].

In the mature CNS, the asymmetrical distribution of Cl^−^ across the membrane (the Cl^−^ inside the cells is maintained relatively low in comparison with the Cl^−^ concentration in the extracellular space, mainly through the action of the potassium-chloride cotransporter 2, KCC2) strongly contribute in defining the reverse potential of GABA_A_-mediated currents (E_GABA_), and it is, therefore, of fundamental importance to guarantee the inhibitory actions of GABA. This also explains why in immature neuronal networks, where the Cl^−^ intracellular concentration is relatively high, GABA can exert excitatory actions [17].

**GABA_B_Rs**

On contrary, GABA_B_Rs are metabotropic, G protein-coupled receptors. They exert their inhibitory action either by decreasing Ca^2+^ currents or by increasing K^+^ conductance [18]. GABA_B_Rs are also expressed at the presynaptic site where by reducing the probability of neurotransmitter release seem to offer a negative feedback mechanism to limit synaptic transmission within a certain physiological range [19].

## 3. Interneuronal Diversity

In the mammalian neocortex, approximately 20%–30% of neurons use GABA as neurotransmitter [20, 21]. 

In contrast to pyramidal cells, GABAergic neurons are an extremely heterogeneous population of cells. Different criteria have emerged in the attempt to classify interneurons based on their different morphological, physiological, and neurochemical features [22], but nonetheless, a universal categorization is still missing. Furthermore, it is extremely difficult to attribute a potentially singular functional role to each subclass [23].

A detailed description of the anatomical and functional properties of different interneurons subclasses is beyond the purpose of this paper; however, it is noteworthy to mention one important structural-functional relation emerging from recent studies: interneurons targeting different domains of principal cells seem to subserve specific functional roles [24]. 

For instance, interneurons preferentially innervating dendrites of principal cells are particularly suited to modulate excitatory postsynaptic potentials (EPSPs) occurring at nearby synapses, thereby limiting the spatiotemporal summation of excitatory inputs and potentially preventing hyperexcitability. Interneurons predominantly sending axons onto the soma and the proximal dendrites (as basket cells) of principal cells are strategically located to control the output of the target neurons, and by operating as a precise clockwork, they can synchronize the firing of large population of principal cells contributing to the generation of cortical oscillatory patterns [25, 26].

## 4. The Effect of Cortical Lesions on GABAergic Transmission

Injuries in the cerebral cortex often lead to an abnormal excitability of the surrounding neuronal networks. An increased spontaneous and evoked neuronal firing has been reported following different experimental models of brain injury [27, 28]. In addition, different functional magnetic resonance imaging (fMRI) studies reported an abnormal activation of commonly silent brain areas in patients suffering from a stroke [29, 30]. 

An increased susceptibility to epileptiform discharges have been observed to progressively develop in lesion models of partially isolated cortex [31–33], in photothrombotic cortical lesion models [34, 35] as well as in patient suffering from brain injuries [5]. 

In parallel, data from many *in vitro *studies revealed a postlesion reduction of the GABAergic intracortical inhibition. This reduced inhibitory transmission was, therefore, considered primarily responsible for the lesion-induced hyperexcitability and for the increased propensity to epileptogenesis.

A reduced strength in the GABAergic intracortical inhibition was reported following a photochemically induced cortical infarct [36, 37] in an experimental model of middle cerebral artery occlusion [38, 39] as well as in the vicinity of a local cortical thermolesion [40] performed in rodents. In these studies, electrophysiological recordings from the surviving neighboring cortical tissue disclosed an impairment of the recurrent intracortical inhibition. 

Beyond this functional evidence, quantitative receptor autoradiography studies have reported a downregulation of radiolabeled muscimol binding to GABA_A_Rs in the surround of a cerebral photothrombosis [28] as well as after unilateral permanent focal cerebral ischemia in the rat brain [41]. The decreased binding of radiolabeled muscimol was interpreted as a reduced density of GABA_A_Rs. 

All together, these studies indicate that cortical injuries, independent of their etiology, can similarly lead to a reduced strength of the inhibitory neurotransmission.

**Time Window**

The lesion-induced suppression of inhibition developed relatively fast, since the effect was already visible one day after the lesion induction [36, 40], it seems to reach a peak in the first week postlesion, and afterwards, it slowly and only partially recovers to a subnormal level two months after the lesion induction [28].

Unfortunately, due to the few chronic investigations, it is still not clear if these relative long-lasting effects are typical of some lesion models and if they depends on the size and location of the cortical damage. Nonetheless, in the subacute phase postlesion (first week postlesion), the impaired inhibition seems to be a phenomenon systematically observed.

**Cellular Mechanisms**

Several cellular mechanisms have been proposed to underlie the lesion-induced suppression of inhibition.

The degeneration of particular vulnerable interneurons subtypes could constitute one plausible mechanism, especially when brain injuries are followed by extensive secondary brain damage. Some studies, performed in models of ischemic and traumatic brain lesions, indeed reported signs of selective suffering and death of interneurons at the border of the injury [42, 43].

GABAergic interneurons could also survive but enter a functional suppress status. 

A large body of evidence demonstrates the existence of a series of modulatory (or homeostatic) mechanisms in the CNS trying to maintain the firing rate of neurons within a certain physiological range in face of dynamic changes in synaptic drive [44–46]. The observed downregulation of the inhibitory strength could be, therefore, seen as a homeostatic mechanism in response to the lesion-induced loss of some excitatory synaptic inputs in the attempt to restore the initial level of neuronal activity. 

Consistent with this hypothesis, a reduction in the number of functional GABAergic synapses has been suggested by several studies. In a lesion model of partially isolated cortex, the structural reconstruction of fast spiking interneurons in the vicinity of the “undercut cortex” revealed a significant reduction in their axonal length and a reduced number of large axonal boutons [47]. At the postsynaptic site, a significant downregulation of the *α*1 and a slight reduction of the *α*2 subunit of GABA_A_Rs were found in the surround of a photochemically induced cortical lesion in rats [43]. Although, based on this finding one cannot rule out a compensatory increase in the expression of others subunits, the parallel decreased binding of radiolabeled muscimol to GABA_A_Rs, observed in another study performed with the same lesion model (see above) suggests an overall reduction in the expression of postsynaptic receptors [28]. 

Furthermore, since the combination of the subunits determines the cellular localization and the functional properties of the GABA_A_Rs [11], even only a shift in the subunits composition, with no change in the expression of the receptors, might profoundly influence GABA-mediated neurotransmission.

Changes in the physiological properties of GABAergic signaling have been also reported. Intracellular recordings from pyramidal cells in the vicinity of an experimentally induced focal cortical infarct [48], in the surrounding of a phototrombotic cortical lesion in rats [3], as well as in a lesion model of partially isolated cortex [49] revealed a positive shift in E_GABA_.

This shift in E_GABA_ toward more depolarized potentials has been primarily attributed to a downregulation of the specific K^+^-Cl^−^ cotransporter 2 (KCC2) with a consequent impaired extrusion of Cl^−^. In support of this hypothesis, some studies performed in traumatic models of axotomized neurons, both *in vitro *and* in vivo,* reported a reduction in KCC2 expression at mRNA and protein level [50, 51]. 

Interestingly, some of the alterations in the GABA-mediated inhibition (e.g., the likely reduced number of GABAergic synapses and the positive shift in E_GABA_) seem to describe a developmental juvenile status when the GABAergic system is still not fully mature.

## 5. Consequences of the Altered Inhibitory Transmission on Cortical Networks Excitability and Functions

Although the association of the reduced GABA-mediated inhibition observed *in vitro* with the hyperexcitability of cortical networks often observed *in vivo* following cortical injuries might seem relatively straightforward, the complexity of the GABAergic signaling and the diversification of interneuronal classes with potential distinct functional roles [22, 23] makes the identification of the underlying cellular mechanisms and the functional consequences on neuronal networks an arduous task. 

Here, bringing together many outstanding studies on neuronal networks function, we provide new elements which will be hopefully helpful in the comprehension of how the altered inhibitory transmission induced by cortical injuries could affect excitability and function of neuronal circuits.

### 5.1. Brain Injury Induced Disturbances in the Excitation-Inhibition (E-I) Balance

In the CNS, the fine-tuned balance between excitatory and inhibitory neurotransmission is essential to guarantee a proper function of neuronal circuits. 

At first glance, such a statement might suggest neuronal transmission to be prone to instability, especially in light of the fact that the excitation-inhibition (E-I) balance is continuously challenged by peripheral stimuli constantly bombarding the CNS.

However, accumulating lines of evidence indicate that in sensory cortices, an increase in excitatory conductance is normally counterbalanced by a similar augmentation of inhibitory conductance [52]. Furthermore, this parallel increase in the level of excitation and inhibition can be maintained over a wide dynamic range conferring to the CNS the capability to respond to a large variation of stimulation intensity without becoming overexcited. 

Despite the substantial flexibility, this dynamic equilibrium can be relative easily compromised by different pathological conditions, such as a brain damage. Different studies performed in animal models of ischemic and traumatic brain injuries indeed reported an important shift in the E-I balance in favour of excitation. Morphological and functional analyses of the rat hippocampus performed few months following a global ischemic episode revealed a dramatic loss of GABAergic presynaptic terminals accompanied by an increase in glutamatergic synapses [53]. Furthermore, in a traumatic brain injury model, recordings from the chronically injured rat sensory-motor cortex did also disclose changes in the efficacy of excitatory and inhibitory neurotransmission in favour of excitation [54]. These anatomical and physiological changes were found to be associated with the onset of epileptic activity indicating a potential important contribution of the shifted E-I balance in the generation of these events.

### 5.2. Recurrent Inhibitory Networks and Potential Consequences of Their Dysfunction

Since the recruitment of recurrent inhibitory circuits plays a key role in the maintenance of the E-I balance, the lesion-induced impairment of intracortical inhibition function [36–39] is most likely one major cause for the development of hyperexcitable neuronal networks. 

However, as already mentioned above, the GABAergic system is extremely heterogeneous, being composed by diverse interneuron cell types with potential specific functional properties [22]. As a consequence of that, the impairment of distinct subpopulations of GABAergic cells will likely have different effects on the excitability and function of cortical networks.

Interestingly, a subclass of dendritic projecting GABAergic neurons expressing somatostatin (SOM neurons) seems to be particularly efficient to counteract increasing levels of cortical excitation. The excitatory synapses impinging on this category of interneurons generate EPSPs which are initially small in amplitude but that progressively increase with the number of subsequent stimuli (facilitating excitatory synapses) eventually leading to the generation of action potentials. This suggests that these cells have the capability to “buffer” a wide range of excitatory inputs before becoming saturated [55], thereby preventing hyperexcitability to occur.

A selective loss of dendritic-projecting SOM containing interneurons has been also reported in human patients [56] and in experimental animal models of temporal lobe epilepsy [57] suggesting a potential involvement of these interneurons in the generation of epileptic seizures. 

Another interneuron subtype which seems to strongly contribute in dampening excessive cortical excitability is constituted by chandelier cells. This category of interneurons, by selectively forming GABAergic synapses onto the axon initial segment of principal cells, can strongly control the generation of action potentials in pyramidal neurons, and therefore, they might have the capability to prevent excessive firing [58]. In line with this assumption, *in vivo* electrophysiological recordings from the somatosensory cortex of rats strongly indicate that chandelier cells do not seem particularly suited to encode incoming ascending information, but they seem indeed strongly involved in preventing hyperexcitability of cortical networks [59]. Furthermore, the selective loss of chandelier cells (or of their axonal terminals) at epileptic foci, reported by different studies, indicates that this cell type might be involved in the generation of epileptic activity [60, 61].

Parvalbumin-containing (PV) basket cells constitute another important class of interneurons strongly participating in the cortical recurrent inhibitory circuits. In distinction to the above-described subtypes of interneurons, PV basket cells seem to strongly participate in the cortical information processing. 

The fast spiking phenotype [62–64], the strong glutamatergic inputs and the short membrane time constant [24] attribute to these interneurons the capability to encode presynaptic inputs with high temporal precision [65]. Furthermore, PV basket cells exhibit strong electrical coupling with each other through gap junctions [66–68] and can finely control the output of pyramidal cells by predominately innervating their perisomatic region [69]. 

Together, these electrophysiological and anatomical properties define the fundamental role of PV basket cells in synchronizing action potential discharges of large numbers of principal cells promoting the emergence of network oscillations in the gamma frequency band (30–80 Hz) [64, 70, 71]. 

Oscillatory activity in the gamma range has been reported to play a crucial role in the perception and processing of sensory stimuli [72], in focusing the attention toward relevant stimuli [73], and in the performance of complex motor actions requiring sensorimotor integration [74]. These findings suggest that the performance of different cognitive tasks requires a physiological function of PV basket cells.

Nowadays, it is still unknown whether the observed reduction of inhibitory transmission following cortical injuries is the result of a lesion-induced effect on a specific subpopulation of inhibitory cells or if all classes of interneurons are equally affected. Potentially, a lesion-induced reduction in the activity of SOM neurons or chandelier cells might critically compromise the E-I balance especially during high level of excitation, while a lesion-induced change in the activity of PV basket cells could have a profound impact on the cortical information processing. 

The recent availability of transgenic mice expressing fluorescent proteins (such as the green fluorescent protein GFP) in defined classes of interneurons [75–77] offers nowadays the possibility to easily investigate how different categories of neurons respond to a cortical lesion, and we are, therefore, confident that in the next years, many of the still open questions will be answered.

### 5.3. Influence of E_GABA_ on the E-I Balance

The reduced strength of the inhibitory transmission, often observed following cortical injuries, does not seem to be simply the result of a lesion-induced degeneration or reduced activity of GABAergic interneurons. The situation is far more complicated, since functional modifications of the GABAergic signaling have been reported after cortical lesions as well. 

An important phenomenon, described following different cortical lesion models, which could potentially compromise the E-I balance, is the positive shift in E_GABA_ [3, 48–51]. 

Generally, GABA is known to exert its inhibitory action by leading to a hyperpolarization of the postsynaptic neuron, thereby driving the membrane potential away from the threshold for the generation of spikes (spike threshold). The positive shift of E_GABA_ at values above the resting membrane potential (*V_m_*) could lead to the straightforward conclusion of an increase in the neuronal excitability due to a GABA-mediated depolarization of the postsynaptic membrane. 

However, depolarization is not always synonymous of excitation [11]. 

GABA_A_-mediated depolarizing responses can still exert an inhibitory action (on conditions that E_GABA_ remains more negative than the spike threshold) by increasing the membrane conductance of the postsynaptic neurons, and thereby shunting excitatory inputs “just” generated at nearby synapses. This inhibitory mechanism, known as “shunting inhibition”, has been shown to operate even under physiological conditions at many cortical and hippocampal synapses, where E_GABA_ was found between the resting *V_m_* and the spike threshold [78–81]. 

From this evidence, one can predict that the pathological positive shift in E_GABA_ might convert many hyperpolarizing GABAergic synapses into shunting ones. 

Nonetheless to estimate the consequences of such a phenomenon on the E-I balance is not an easy task. Shunting inhibition can have in some instances a stronger inhibitory effect than hyperpolarization. This is because at depolarized membrane potentials GABA_A_Rs exhibit a higher ionic conductance (or outward rectification) [16, 82]. Moreover, shunting inhibition cannot lead to the opening of hyperpolarization-activated cation channels and does not favour the deinactivation of voltage sensitive sodium and low threshold calcium channels as hyperpolarizing postsynaptic potentials do [83]. For these reasons, shunting inhibition can prevent the generation of “rebound excitation” in some neurons [11]. 

However, on the other side, the efficacy of shunting inhibition is strictly dependent on how the excitatory and inhibitory inputs are spatially and temporally related on the membrane of the postsynaptic cell [84]. Temporally, excitatory glutamatergic inputs can be maximally attenuated when shortly preceding the activation of neighbouring shunting inhibitory postsynaptic potentials (IPSPs). Spatially, shunting IPSPs close to the soma of the cells can better control the integration of excitatory depolarizing inputs coming from distal dendrites. 

In contrast, IPSPs temporally and spatially isolated from EPSPs need to be hyperpolarizing to provide an inhibitory action; otherwise, they will generate depolarizing waves propagating toward the soma of the cell which will sum to depolarizing EPSPs [85]. A pathological shift in E_GABA_ at these synapses could, therefore, critically favour hyperexcitability of cortical networks.

Finally, if the depolarized E_GABA_ is due to an impaired Cl^−^ extrusion, as suggested by Jin and colleagues, repetitive synaptic GABA_A_Rs activation, normally occurring *in vivo*, could promote a transient additional intracellular Cl^−^ accumulation which will depolarize E_GABA_ further until the action of GABA will be fully excitatory [49]. This hypothetical transient depolarized shift in E_GABA_ could facilitate recurrent excitation between pyramidal cells potentially leading to the generation of epileptic discharges. 

All these considerations explain why it is difficult to predict the consequences of the reported lesion-induced depolarized E_GABA_ on the excitability of neuronal networks. Nonetheless, if E_GABA_ is equally affected at all synapses, a general shift in favour of excitation should be expected.

### 5.4. Increase in Tonic Inhibition Postlesion

Despite the large number of studies strongly indicating a postlesion impairment in GABA-mediated inhibition, a recent study performed in a phototrombotic model of stroke in the motor cortex of mice, revealed a lesion-induced enhancement of tonic inhibition due to an increased activity of GABA_A_Rs containing the subunit *α*5 and *δ* in the peri-infarct cortex [86]. GABA_A_ receptors containing these subunits are normally located extrasynaptically [12, 87], where they can be tonically activated by low concentration of GABA in the extracellular space (ambient GABA) leading to the generation of a tonic conductance in the postsynaptic neuron [15]. The authors reported that the enhanced GABA-mediated tonic inhibition was due to an increased ambient GABA as a consequence of an impaired GABA uptake from the astrocytic GABA transporters, GAT-3/GAT-4.

Interesting, in a study performed in the hippocampus of guinea pigs tonic inhibition was found to be most prominently expressed at interneurons [88]. Assuming a similar scenario in the neocortex, the excessive tonic inhibition postlesion might strongly suppress interneurons activity leading to a decrease in the GABAergic synaptic transmission. 

A cortical lesion may, therefore, produce a shift from a phasic to a tonic GABAergic transmission with profound consequences on neuronal network functions [86].

Tonic inhibition lacks the spatial and temporal precision of synaptic transmission, and by producing a “long-term” reduction in the resistance of the postsynaptic neurons, it could prevent an appropriate neurotransmission, potentially constraining plastic processes to occur. Moreover, the enhanced tonic inhibition might also contribute to the above-mentioned depolarizing shift in E_GABA_ by promoting intracellular Cl^−^ accumulation (especially if the rate of Cl^−^ influx, through tonically active extrasynaptic GABA_A_Rs, overcomes the function of the Cl^−^ extruder KCC2).

### 5.5. Changes in the GABAergic Transmission in Brain Areas Remote to the Injury

The functional consequences of neocortical injuries are often not limited to the neuronal circuits surrounding the primary lesion but can be observed in remote projection cortical areas as well as in some subcortical structures [89]. These remote alterations in brain function following a focal brain damage are known as “diaschisis” and were firstly described by von Monakov as early as in the 1914. He suggested that these remote effects must be likely attributed to the deafferentiation of damaged fibers from the injured area. Nowadays, the term “diaschisis” is used by many authors to describe acute and chronic changes in cerebral blood flow, metabolism, and electrical activity in remote areas following brain lesions. Particularly interesting is the frequently observed “transhemispheric diaschisis” following unilateral lesions in the cerebral cortex likely due to the deafferentiation of transcallosal connections from the damaged area [1]. 

In different clinical studies [29, 30] and experimental animal models of stroke [90], this “transhemispheric diaschisis” has been described as an abnormal increase in the activity of the cortical hemisphere contralateral to the lesion.

In parallel, *in vitro* extracellular recordings performed in photothrombotic and ischemic unilateral cortical lesion models [37–39] revealed a reduced strength in the GABAergic transmission widespread throughout the intact contralateral hemisphere. 

It is, therefore, conceivable that the reduced inhibitory tone may be responsible for the described abnormal activation of the hemisphere contralateral to the lesion. 

As a consequence of these findings, the hypothesis emerged that a lesion-induced dysinhibition of the contralateral cortex could potentially contribute to the functional recovery postinjury by compensating or at least partially taking over the function of the damaged brain area. 

In this regard, longitudinal studies, comparing the extent of the hyperexcitability of the unaffected cortex with the degree of recovery from neurological deficits, suggested that the abnormal activity of the contralateral hemisphere during the acute/subacute phase postlesion could indicate a sort of bihemispheric cooperation which might be indispensible for performing even simple tasks involving the affected side of the body. However, the contribution of the contralateral cortex, in the recovery of function, seems to diminish over time, since the better final outcomes are observed when the brain regions, normally executing a function, are reintegrated into the active network [91]. 

It is, therefore, plausible that shortly after a focal cortical injury, the dysinhibition of anatomically connected remote areas might constitute a compensatory mechanism to temporary relieve the neurological deficits before a consistent functional reorganization will gradually guaranty a permanent, at least partial, functional recovery. However, it is also not possible to rule out a potential involvement of these hyperexcitable remote neuronal networks in promoting the generation of epileptic events after a brain injury.

### 5.6. Lesion-Induced Alterations of Thalamocortical Activity as Potential Source of Hyperexcitability

Since the brain areas most likely affected by a cortical damage are the one anatomically connected to the lesion site, the dense corticothalamic thalamocortical connections strongly suggest a likely lesion-induced physiological alteration at the level of the thalamus. One study performed in a phototrombotic model of cortical infarct in the somatosensory cortex of rats indeed reported a strong reduction in the excitability of interneurons located in the reticular thalamic nucleus [92]. The reticular thalamic nucleus is constituted by GABAergic interneurons which, by receiving the main excitatory drive from the cortex and providing inhibition onto thalamocortical relay cells, can strongly modulate the thalamocortical flow of information [93]. The authors suggested that the dysfunction of this inhibitory thalamic nucleus might produce a powerful dysinhibition of thalamocortical activity which could be potentially involved in the generation of postlesion epileptiform activity. Consistently, dysfunctions of the thalamocortical circuitry have been already implicated in the genesis of generalized epilepsy [94–96]. 

It is, therefore, important, when searching for the cellular mechanisms responsible for epilepsy after cortical injuries, to do not underestimate potential alterations in the physiological properties of thalamic neurons.

## 6. The Influence of GABAergic Transmission on Neuronal Network Plasticity, the Other Side of the Coin

From the considerations drawn so far, it seems pretty evident that the pathological alterations of GABAergic inhibition following a cortical lesion can lead to severe negative consequences on the excitability and function of neuronal circuits. One might, therefore, conclude that a simple pharmacological enhancement of the GABAegic synaptic transmission could be the best approach to restore a normal brain activity after a lesion. 

However, the reduced inhibition could be also viewed as an evolutionary conserved mechanism initiated in front of a dramatic alteration of cortical activity, as in the case of cortical injuries, with potential beneficial effects.

In line with this assumption, different studies indicated that the level of intracortical inhibition is important to define the plastic properties of neuronal circuits.

### 6.1. The Influence of the Level of Cortical Inhibition on Neuroplasticity

During the development of the mammal CNS, the slowly increasing strength of inhibitory transmission is suggested to modulate cortical plasticity by crossing two thresholds: crossing the first threshold defines the onset of a period, known as critical period, characterized by high experience-dependent plasticity of neuronal networks, while passing the second threshold closes the time window of high plasticity and open a period of restricted plasticity which is protracting throughout the life of an animal [97, 98]. 

The relation between the level of intracortical inhibition and the critical period for plasticity has been extensively studied in the visual cortex of rodents, where the critical period is normally determined by the successful induction of ocular dominance (OD) plasticity [99]. In this system, a developmental modulation of the GABAergic strength, achieved by combining pharmacological and genetic tools, has been shown to be effective in shifting the onset and closure of the OD plasticity [100, 101]. Furthermore, in a recent study, Harauzov and colleagues could demonstrate that the pharmacological reduction of a mature GABAergic inhibition was sufficient to trigger the reactivation of the OD plasticity in the visual cortex of adult rats [10]. This finding suggests that even a simple functional modification of inhibition could be enough to modulate the plastic properties of neuronal networks. 

Based on these observations, if the reduced strength of inhibition observed after a cortical lesion matches a level similar to that found during the critical period, the remodeling capacity of the surrounding cortical networks could be strongly enhanced.

### 6.2. GABA-Mediated Inhibition as a “Filter” for Plasticity at Excitatory Inputs

In order to take advantage of the described findings, it is fundamental to elucidate the cellular and physiological mechanisms mediating the influence of the strength of inhibition on cortical plasticity.

As early as in 1987, Artola and Singer proposed that strong inhibitory synapses, by reducing EPSPs, could prevent the activation of NMDARs, indispensible for many forms of synaptic plasticity [102].

Shortly afterwards, Kirkwood and Bear also suggested that a mature inhibitory circuitry in layer 4 of sensory cortices might act as a kind of filter by limiting the activity pattern able to gain access from subcortical structures to the supragranular layers of the cortex [8].

This observation indicates that inhibition may control plasticity of neuronal networks by selectively permitting or preventing plasticity at excitatory synapses. 

A reduced/immature GABAergic transmission might, therefore, act as a permissive substrate allowing sensory experience to remodel structure and functions of cortical networks. However, a too drastic impairment of synaptic GABAergic transmission might be deleterious by preventing accurate cortical information processing and by promoting epileptic activity. On this concern, Feldman proposed the existence of an ideal level of inhibition, on the one hand low enough to permit the potentiation or the depression of excitatory connections but on the other hand sufficient to guarantee an appropriate temporal encoding of relevant inputs [97].

Reducing the strength of inhibition or adjusting an impaired inhibition postlesion to an ideal level could, therefore, constitute a promising tool to restore and/or enhanced experience-dependent plastic processes in the adult CNS. 

Among the different interneuron subtypes, PV basket cells have been suggested to contribute, more than others, in the expression of the critical period for cortical plasticity [103, 104]. 

Different functional properties of PV basket cells can indeed support their role in modulating plastic processes. Their fast somatic inhibition could filter the action potentials able to access the dendritic arbor by back propagation, thereby allowing postsynaptic spikes to meet presynaptic inputs within specific temporal windows appropriate for synaptic plasticity induction [105]. Furthermore, PV basket cells, being electrically coupled through gap junctions (see above) are able to detect strong synchronous activity arriving in the cortex, which normally carries relevant information from the periphery [106]. These interneurons are, therefore, well suited to produce competitive outcome by reinforcing relevant and favouring the elimination of irrelevant connections based on the sensory experience [98].

### 6.3. Plasticity of Inhibitory Circuits

Beside permitting or preventing structural and functional modifications of excitatory connections, inhibitory networks can themselves undergo plastic processes. 

Firstly, the activity of cortical interneurons is highly sensitive to global changes in the activity of cortical circuits. A reduced cortical activity leads normally to an impaired GABAergic innervation [107, 108] and to a decreased GABAergic neurotransmission [109–111]. This activity-dependent modulation of inhibitory strength seems to be an important homeostatic mechanism playing a crucial role in the maintenance of a proper E-I balance [46]. 

Not only homeostatic but also Hebbian plastic mechanisms have been observed at inhibitory synapses. Several *in vitro* studies could demonstrate the efficacy of different stimulation protocols in the induction of long-term modifications at cortical GABAergic synapses [112–114]. 

Moreover, some *in vivo* studies performed in different sensory systems provided evidence of inhibitory-plasticity-dependent changes in cortical maps. 

In these studies, a shift in cortical maps was obtained by exposing the animals to an abnormal sensory experience for a define period of time. This condition produced a receptive field shift away from deprived/inappropriate inputs towards new behavioral relevant inputs. Interestingly, this receptive field plasticity could be reversed by the application of a GABA_A_Rs blocker indicating that the suppression of responses to irrelevant inputs was likely due to a potentiation of GABAergic synapses [115, 116].

### 6.4. Influence of Intracortical Inhibition on Cortical Map Plasticity

Intracortical GABA-mediated inhibition also strongly contributes in shaping the receptive fields of cortical neurons.

This important function of GABA was first appreciated in a series of electrophysiological studies mainly performed in primary sensory systems of mammals. In these studies, the application of a GABA_A_Rs antagonist produced an enlargement of the receptive field's size of single neurons [117, 118] as well as profound alterations in the receptive field properties such as the loss of orientation and direction selectivity in neurons of the visual cortex [6, 119] and a dramatic expansion of tuning curves in the auditory cortex [120]. 

Remarkably, increased and/or altered receptive fields were observed following cortical lesions in the surrounding brain areas [121–123]. 

The lesion-induced reduction of inhibition might enlarge the receptive fields by bringing suprathreshold and thereby unmasking previously silent (subthreshold) inputs [124].

Converting silent connections into functional ones is *per se* a mechanism of functional reorganization, but most importantly, as already outlined above, this process may allow new functional excitatory inputs to enter in competition with others and to undergo potentiation or depression following Hebbian-based learning rules. 

In this way, a reduced level of inhibition could strongly contribute in the plasticity of cortical maps.

### 6.5. A Cellular Model of Functional Reorganization Following Cortical Injuries

The above-mentioned studies provide several lines of evidence for an important role of inhibition in influencing the plasticity of cortical networks. 

Here, we briefly discuss how the plasticity of neuronal networks surrounding a cortical lesion could mediate the recovery of function and how the lesion-induced reduction in inhibition could contribute to this process. 

First of all, to achieve a functional recovery after a cortical lesion the information previously processed by the injured cortex needs somehow to be rerepresented by the remaining cortical areas.

**Figure 1:**
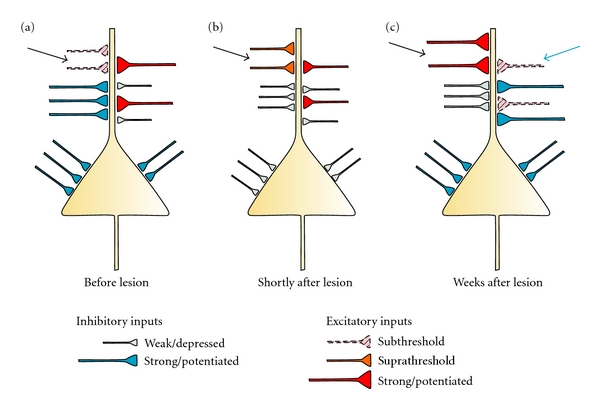
Schematic illustration representing a model pyramidal neuron in the cortex surrounding the lesion with its excitatory and inhibitory inputs before, shortly after and some weeks after the lesion occurrence. This model shows the potential cellular mechanisms responsible for the functional rewiring of neuronal networks following cortical injuries. (a) Before the occurrence of a cortical lesion, some excitatory inputs are subthreshold (arrow) being masked by strong inhibitory inputs; (b) early after the cortical lesion occurrence (first week postlesion), subthreshold connections can be converted into functional (suprathreshold) ones (arrow) by the lesion-induced weakening of inhibitory inputs; (c) some weeks after the lesion, experience-dependent plastic processes will likely lead to the reinforcement of some of the new functional inputs, which turn out to be behavioral relevant after the lesion (black arrow) and to the suppression of excitatory inputs which became irrelevant (blue arrow). For clarity, many cellular and subcellular elements have been omitted; this draw represents, therefore, an oversimplification of a real scenario.

In the neocortex, due to the dense and exuberant cortical connectivity [125], some of the normally silent connections projecting onto surviving neurons could be anatomically capable of transmitting information previously process by the damaged tissue (Figure 1(a)). Remarkably, these silent connections can be converted into functional ones by the extensively described lesion-induced reduction of inhibitory neurotransmission (Figure 1( b)).

The initial depression of GABAergic inputs, by unmasking subthreshold excitatory connections, plays therefore a crucial role in the initiation of cortical map plasticity.

This is of fundamental importance since cortical map plasticity is largely responsible for the “long-term” functional recovery postlesion. 

Subsequently, to guarantee a stable rewiring of neuronal circuits, experience-dependent plastic processes will likely lead to the reinforcement of some of these new functional inputs, which turn out to be behaviorally relevant, and eventually lead to the suppression of inputs which became irrelevant after the lesion (Figure 1(c)).

The reinforcement of the relevant inputs most likely involves long-term potentiation (LTP) of excitatory connections [126–128], while improper inputs could be masked by potentiated inhibitory connections [115, 116] or may directly undergo long-term depression (LTD) [129]. 

Finally, structural modifications might stabilize the new connectivity patterns.

This process can therefore induce at least a partial functional recovery postlesion, because it promotes the cortical area surrounding the damage to gradually take over the functions before belonging to the death cortical tissue.

## 7. Strategies toward a Better Functional Recovery Following Cortical Injuries

As we intensively discussed, the altered GABA-mediated inhibition often observed following cortical injuries can have both detrimental consequences by modifying excitability and functions of cortical networks as well as beneficial effects by promoting cortical plasticity. 

Intuitively, in order to optimize the functional recovery of patients suffering from a cortical injury, a therapy should aim to minimize the deleterious consequences of a modified inhibitory transmission without preventing the potential beneficial effects on cortical plasticity. 

This scope can be achieved if we could distinguish that the positive and negative effects of the altered GABAergic transmission differ somehow in the cellular mechanisms of their induction, in the GABAergic networks that they affect and/or in the temporal window postlesion of their expression.

Although much more needs to be done to give a final answer to these questions, a consistent amount of information can already be found in many studies investigating the role of GABA-mediated inhibition on cortical function and plasticity. 

For instance, in the above-mentioned study of Clarkson and colleagues it has been proposed that the stroke-induced increase in the tonic GABAergic transmission is one of the constraining factors for cortical plasticity. The authors were indeed able to demonstrate that dampening the excessive tonic inhibition, by selectively antagonize the function of extrasynaptic GABA_A_Rs, produced a significant improvement in the motor recovery of the animals [86]. 

The availability of antagonists for specific GABA_A_Rs subunits exclusively or predominately contained in extrasynaptic GABA_A_Rs [130] makes the selective reduction of tonic inhibition a plausible tool to improve the functional recovery of patients suffering from a cortical lesion.

The identification of specific networks of GABAergic neurons primarily involved in the reorganization of cortical circuits postlesion could also promote the development of a better targeted therapy to improve the functional recovery of patients. On this regard, although it is still not possible to attribute an exclusive function to each subtype of cortical interneurons, compelling evidence indicates that some classes of GABAergic cells might be more relevant than others in mediating cortical plastic processes.

In particular, through the lines of this paper different points stress the importance of the fast-spiking PV basket cells in the regulation of cortical network functions as well as in the modulation of experience-dependent plastic processes. A drastic impairment in the function of these interneurons should be, therefore, avoided although a moderate reduction in their activity might facilitate cortical plastic processes. 

On contrary, other subtypes of interneurons seem to contribute to a lesser extent in the induction of cortical plasticity and to be more closely involved in controlling the excitability of cortical networks. On the basis of recent findings, these subpopulations might include dendritic projecting SOM interneurons and chandelier cells [55, 59, 104]. Preventing a drop in the activity of these interneuron subtypes could constitute a neuroprotective tool against the development of postlesion epileptic discharges, while a lesion-induced moderate reduction in the activity of PV basket cells might be better tolerate and could even offer enhanced plastic properties to the surviving cortical tissue. 

Interestingly, PV basket cells form predominately perisomatic synapses enriched in *α*1 subunit-containing GABA_A_Rs [131], while other interneurons subtypes, as in particular chandelier cells, formed synapses enriched in *α*2-containing GABA_A_Rs [132].

The development of pharmacological agents showing specific-subunit sensitivity might, therefore, provide a strategic tool able to modulate the function of a particular class of interneurons and might be more effective in reducing postlesion cortical hyperexcitability without constraining cortical plasticity. 

Since experience-dependent changes in synaptic plasticity likely contribute to the functional rewiring of cortical networks, a physical rehabilitation accompanying a pharmacological approach will remain essential. 

Finally, the identification of an optimal therapeutical time window for pharmacological and rehabilitative interventions could also be extremely helpful. 

In this regard, results from clinical studies indicate that pharmacological therapies following cortical injuries showed a moderate efficacy and only if administered very early after the lesion (few hours postlesion) [133]. This might be due to the fact that so far the largest effort has been dedicated in the development of a neuroprotective tool to prevent or reduce the secondary brain damage. 

Now, it seems that the attention is shifting toward the development of a therapy aiming to amplify endogenous mechanisms of repair [134]. This might produce better functional outcomes and could offer a prolonged temporal window of intervention potentially extending into the subacute and chronic phase postlesion.

As extensively described in this paper this time window postlesion seems to be characterized by a profound alterations in the GABAergic transmission which might strongly influence cellular mechanism of neuroplasticity. A therapeutical approach able to precisely target the GABAergic signaling involved in the modulation of neuronal plastic processes may, therefore, constitute a powerful instrument to improve the rehabilitation of patients suffering from traumatic brain injuries and stroke.
